# Enhancing the Health-Promoting Effects of Tomato Fruit for Biofortified Food

**DOI:** 10.1155/2014/139873

**Published:** 2014-03-12

**Authors:** Assunta Raiola, Maria Manuela Rigano, Roberta Calafiore, Luigi Frusciante, Amalia Barone

**Affiliations:** Department of Agricultural Sciences, University of Naples “Federico II”, Via Università 100, Portici, 80055 Naples, Italy

## Abstract

Consumption of tomato fruits, like those of many other plant species that are part of the human diet, is considered to be associated with several positive effects on health. Indeed, tomato fruits are an important source of bioactive compounds with known beneficial effects including vitamins, antioxidants, and anticancer substances. In particular, antioxidant metabolites are a group of vitamins, carotenoids, phenolic compounds, and phenolic acid that can provide effective protection by neutralizing free radicals, which are unstable molecules linked to the development of a number of degenerative diseases and conditions. In this review, we will summarize the recent progress on tomatoes nutritional importance and mechanisms of action of different phytochemicals against inflammation processes and prevention of chronic noncommunicable diseases (e.g., obesity, diabetes, coronary heart disease, and hypertension). In addition, we will summarize the significant progress recently made to improve the nutritional quality of tomato fruits through metabolic engineering and/or breeding.

## 1. Introduction

The tomato belongs to the Solanaceae family that includes more than 3,000 species. In particular, the section of the* Lycopersicon* genus* Solanum* consists of 13 species or subspecies: the cultivated tomato,* Solanum lycopersicum*, which is the only domesticated species, and 12 wild species (such as* S. chmielewskii*,* S. habrochaites*,* S. pennellii,* and* S. pimpinellifolium*). Tomato fruits are an important source of nourishment for the whole world's population. Its world production is estimated to be around 159 million tons, while the average annual fresh tomato consumption is 18 kg per European and 8 kg per capita in the US [1]. In the last few years, tomato consumption has further increased since tomato fruits supply both fresh market and processing products such as soups, juices, purees, and sauces. The Economic Research Service of the USDA estimates that 35% of raw tomatoes are processed into sauces, 18% into tomato paste, 17% into canned tomatoes, 15% into juices, and 15% into catsup [2]. The tomato fruits, like those of many other plant species that are part of our diet, are an important source of substances with known beneficial effects on health, including vitamins, minerals, and antioxidants [3]. Indeed, tomato fruit consumption has been associated with a reduced risk of inflammatory processes, cancer, and chronic noncommunicable diseases (CNCD) including cardiovascular diseases (CVD) such as coronary heart disease, hypertension, diabetes, and obesity [2]. Antioxidant metabolites are a group of vitamins, carotenoids, phenolic compounds, and phenolic acid, with health-enhancing effects on our body [2, 3]. The total antioxidant activity of tomato fruits is commonly classified into hydrophilic and lipophilic ones. The first is conferred mainly by soluble phenolic compounds and vitamin C and shows a significant impact on total antioxidant activity (83%), while the latter is conferred by carotenoids, vitamin E, and lipophilic phenols (17%) [4]. Many factors such as genetics (cultivar or variety), environment (light, temperature, mineral nutrition, and air composition), and cultural practices (ripening stage at harvest and irrigation system) affect the chemical composition of tomatoes [5, 6]. In this paper, a global point on tomatoes nutritional importance and mechanisms of action of different phytochemicals against inflammation processes according to the last discoveries is discussed.

Recently, significant progresses have been made in order to improve the levels of human health promoting compounds in tomato fruits through metabolic engineering and/or breeding [7, 8]. However, breeding work is complicated by the complex nature of many of these characters, which often requires quantitative genetic approaches for the identification of genes and QTLs (quantitative trait loci) involved in their regulation [9]. In fact, the synthesis of many of the compounds responsible for the tomato nutritional quality is the result of coordinated activities that involve many of the primary and secondary metabolism pathways regulated by developmental, physiological, and environmental signals.

Therefore, in this paper, we will summarize the recent progress made to improve the nutritional quality of tomato fruits according to current state of the art.

## 2. Bioactive Tomato Compounds 

Regular consumption of tomato fruit and its products is associated with lower risk of CNCD and several types of cancer and inflammation because of interaction of phytochemicals with metabolic pathways that are related to inflammatory response and oxidative stress.

Some evidences support a role for tomato products in the prevention of lipid peroxidation that is a risk factor of atherosclerosis and cardiovascular disease [10].

High dietary intake of tomato products can reduce LDL cholesterol levels and increase LDL resistance to oxidation [11]. In addition, Burton-Freeman et al. [12] reported that tomato consumption during a meal attenuated postprandial lipemia-induced oxidative stress and associated inflammatory response. An important role of tomatoes has been also attributed to the conservation of DNA stability. Daily intake of a tomato drink called Lyc-O-Mato significantly reduced (by about 42%) DNA damage in lymphocytes subjected to oxidative stress [13]. These effects are related to the presence of several phytochemicals in raw tomatoes and their products.  Table 1 shows the general effects of tomato bioactive compounds on health, while Table 2 summarizes the main evidences about anti-inflammatory and antioxidant activities.

### 2.1. Carotenoids

Carotenoids are tetraterpenes belonging to fat-soluble pigments. They include provitamin A carotenoids, such as *β*-carotene and *β*-cryptoxanthin, and non-provitamin A carotenoids, such as lutein and lycopene. They contribute to the photosynthetic machinery in plants and protect them against photo damage [67]. More than 600 carotenoids were identified in nature, among which around 40 are present in food normally included in human diet. [68]. Geographical location has a great impact on carotenoid content and bioaccessibility. For example, carotenoid content of various tomato varieties grown in Ireland differed from those grown in Spain [69].

Several studies have reported the health benefits of carotenoids related to their antioxidant power, such as immune system stimulation and antitumor activity [18, 45, 70]. Dietary supplements of carotenoids may act as moderate hypocholesterolemic agents, as a consequence of their inhibitory effect on macrophage 3-hydroxy-3-methyl glutaryl coenzyme A (HMGCoA) reductase, the rate-limiting enzyme in cholesterol synthesis [71]. Carotenoids cause changes in the expression of many proteins participating in cell proliferation and signaling pathways. For example, lycopene is associated with reduction of cyclin D1 protein, a known oncogene overexpressed in many primary tumors. In addition, lycopene can increase expression of several differentiation-related proteins, such as cell surface antigen (CD14), oxygen burst oxidase, and chemotactic peptide receptors [72]. According to scientific evidences, the carotenoid intake from tomatoes is the most important nutritional contribution of this fruit, and therefore, the highest weight in the index of antioxidant nutritional quality is attributed to carotenoids content [3].

#### 2.1.1. Lycopene

Tomatoes contain 8–40 *μ*g per gram fresh weight of lycopene, about 80% of total dietary intake of this carotenoid [73, 74]. Field-grown tomatoes appear to contain higher levels of lycopene, ranging from 5.2 to 23.6 mg/100 g fresh weight (FW) than greenhouse-grown tomatoes (0.1 and 10.8 mg/100 g FW) [75]. Accumulation during fruit ripening results from the downregulation of the lycopene cyclase gene (*CrtL*), a gene conserved through evolution from the time of cyanogenic bacteria [76]. Lycopene is a polyunsatured molecule containing 13 double bonds that can exist in* trans*- and* cis*-configurations [77]. In fresh tomatoes, lycopene is mainly found in* trans*-conformation, while thermal treatments, light, acids, oxygen, and digestion can cause transformation into the more bioactive* cis*-form [78]. Lycopene is the main phytochemical in tomato fruits for its strong antioxidative role associated with its ability to act as free radical scavengers from reactive oxygen species (ROS), generated by partial reduction of oxygen [79]. Furthermore reactive nitrogen species (RNS) can be produced. ROS and RNS include free radicals and other nonradical reactive derivatives also called oxidants [80]. They are produced either from normal cell metabolisms* in situ* or from external sources (pollution, radiation, and cigarette smoke). Radical accumulation in the body generates a phenomenon called oxidative stress that results from an imbalance between formation and neutralization of ROS/RNS in cells [81]. One of the most undesirable effects of ROS is lipid peroxidation with consequent formation of radicals. The products formed during these mechanisms are toxic. In particular malondialdehyde can cause mutagenic lesions [82]. These processes play a major role in the aging and development of chronic and degenerative illness such as cancer, atherosclerosis, autoimmune disorders, cataract, rheumatoid arthritis, and neurodegenerative and cardiovascular diseases. Lycopene can transform the high reactive free electron on DNA to a more stable free radical by delocalization along its conjugate 13 double bonds.

ROS can activate the transcription nuclear factor kappa B (NFkB) that gives rise to the expression of genes of pro- and anti-inflammatory cytokines and their subsequent production [83]. It is demonstrated that lycopene inhibits this activation [14, 15]. Anti-inflammatory cytokines such as IL-10 are produced to control inflammation, while proinflammatory cytokines including tumor necrosis factor-alpha (TNF-*α*), IL-6, and IL-8 increased the inflammatory response. Lycopene stimulates the first one and inhibits the latter in macrophages and adipocytes [84]. Doses of 10 mg lycopene/day administered for 3 months decreased PSA (Prostate Specific Antigen) level, tumor grade, bone pain, and urinary tract symptoms in patients with metastatic prostate cancer [19], interfering with growth factor receptor signaling and cell cycle progression [85]. In addition, lycopene can inhibit other cancer types (breast, colorectal, endometrial, lung, oral, and pancreatic) [18]. Nevertheless, according to some studies, lycopene is not potent enough to be clinically useful as a drug for prostate cancer because high concentrations (above 1 *μ*mol/L) are needed to achieve significant response in humans [86–88]. In presence of diseases such as cancer and cardiovascular diseases, higher levels of lycopene ranging from 35 to 75 mg per day may be required [89]. Lycopene can modulate cyclooxygenase pathways and xenobiotic metabolism because *β*-carotene and lycopene supplementation interact with the metabolism of linoleic acid, resulting in either an increase (*β*-carotene) or decrease (lycopene) in plasma concentration [71]. An 83% reduction in prostate cancer risk was observed in a group of men enrolled in the Physician's Health Study, with the highest plasma lycopene concentration (0.40 *μ*m/L) in comparison with the lowest lycopene group (0.18 *μ*m/L) [90].

Many chronic diseases and increasing of mortality are directly related to the global obesity epidemic (body mass index ≥30 kg/m^2^), which started in the 1980s. Adolescent populations are currently the group mainly exposed to risk of obesity [91]. Serum concentrations of C-reactive protein (CRP), tumor necrosis factor (TNF-*α*), and interleukin (IL)-6 are significantly correlated with weight, body mass index (BMI), and waist circumference [92]. These aspects assume a major importance considering that a link between skeletal muscle and adipose tissue has been reported and related with the control of body weight, muscle mass, and fat mass. In particular interleukin (IL)-15 and TNF-*α* play an important role in crosstalk between adipose tissue and skeletal muscle [93].

IL-6 and TNF-*α* contribute to liver production of CRP and atherosclerosis by inducing insulin resistance and upregulating the expression of other inflammatory mediators. Di Renzo et al. [94] reported that, in Italian Caucasian females, the fat mass percentage (FM%) is a main factor of an increase in IL-6 production and insulin resistance. In particular, −174 G/C polymorphism in IL-6 promoter has been recognized as a marker which could contribute to identify vulnerable individuals at risk of age and obesity-related diseases.

There is now strong evidence for the benefits of tomatoes in terms of protection against diseases associated with metabolic syndrome and obesity.

In particular, lycopene reduces transcript levels of proinflammatory cytokines with downregulation of IL-6 expression [16, 17]. Markovits et al. [16] reported the effect of tomato-derived lycopene consumption on markers of inflammation and oxidative stress in a group of patients with extreme obesity. Obese patients showed abnormally higher markers of inflammation and oxidation products and lower plasma carotenoids compared to control subjects (0.54 versus 0.87 *μ*g/mL). Following lycopene treatment, a significant elevation of plasma carotenoids, specifically lycopene, occurred in the treatment versus the placebo group. Kuopio Ischemic Heart Disease Risk Factor Study examined the relation between serum antioxidant and intima-mediated thickness of the common carotid artery, a marker related to the risk of having an acute coronary event. Lower levels of plasma lycopene were showed in men who had a coronary event compared with men who did not [17]. Several studies demonstrated that anti-inflammatory effects derived by tomato products consumption were superior to that of lycopene delivered as a single compound [27, 95].

#### 2.1.2. *β*-Carotene

*β*-Carotene is considered a provitamin because it can be converted into retinol, a compound essential for vision. It is known as a strong antioxidant and the best quencher of singlet oxygen. At low partial pressures of oxygen, such as those found in most tissues under physiological conditions, *β*-carotene was found to inhibit oxidation. In tomatoes grown in a field located in the S. Marzano area, reported values were between 0.28 and 1 mg/100 g FW [3]. These levels were in accordance with other studies [96, 97]. In commercial cherry tomato varieties, *β*-carotene amount reached 1.2 mg/100 gr FW [98]. Bioaccessibility of *β*-carotene from raw tomatoes was reported to be about 0.1% [99].

A study found that both *β*-carotene and lycopene (2.5 mmol/L, 2 h) totally abolished TNF-*α*-induced ROS levels (1 ng/mL, 16 h) in TNF-*α*-treated human umbilical vein endothelial cells. This was due to the redox balance protection and to the maintenance of nitric oxide (NO) bioavailability [59]. Several studies demonstrated that *β*-carotene prevents photooxidative damage and sunburn showing photoprotection in humans. Erythema formation was significantly diminished when *β*-carotene was applied on human skin or with a dietary intervention alone or in combination with *α*-tocopherol for 12 weeks [20]. Metabolites derived from all-*trans*-*β*-carotene inhibit atherosclerosis in hypercholesterolemic rabbits. This could be due to stereospecific interactions with retinoic acid receptors in the artery wall [100].

Recently, Karppi et al. [21] found that low concentrations of serum *β*-carotene may be associated with an increased risk of CHF (congestive heart failure) and cardiac death in men.

However, some studies do not support the absolute favorable role of *β*-carotene assumption. The initial antioxidant activity of *β*-carotene is followed by a prooxidant action at high oxygen tension. This mechanism may be related to adverse effects observed under the supplementation of high doses of *β*-carotene. A study reported that supplements in doses of 20 mg daily for 5–8 years had no protective effect on macrovascular outcomes or total mortality of diabetic male smokers [101]. Margalit et al. [24] reported that there was no significant difference between risks of lethal prostate cancer with the use of *β*-carotene during radiation therapy compared with that of placebo. Other trials about *β*-carotene supplementation will be required in order to clarify the protection role associated with *β*-carotene.

#### 2.1.3. Lutein

Lutein is a yellow carotenoid synthetized in chloroplasts and chromoplasts and found in high quantities in leaves whose main function is to contribute to photosynthesis [102, 103]. It is one of the most widely found carotenoid xanthophyll pigments in fruits and vegetables normally consumed. Average lutein concentration in raw tomato was reported up to 32 *μ*g/100 g FW [104], while Guil-Guerrero and Rebolloso-Fuentes [105] reported a content up to 800 *μ*g/100 g FW in cherry variety. Increasing interest around this compound was due to its important role in preserving eye health in association with zeaxanthin [106]. The improvement of visual function and symptoms was also reported in atrophic age-related macular degeneration (ARMD), a condition including genetic, cardiovascular, nutritional, and environmental factors [22, 23]. Nevertheless, up to date, the relationship between the consumption of lutein and maintenance of normal vision has not been clearly demonstrated [107].

Epidemiological studies* in vitro* and in mouse model demonstrated that increased dietary intake of lutein is protective against the development of early atherosclerosis in human and animals through the reduction of inflammation and oxidative stress in the artery wall [46]. Kabagambe et al. [108] and Riccioni et al. [25] reported that there was an inverse correlation between the risk of developing a myocardial infarction, adipose tissue lutein content, and dietary lutein intake. Furthermore, carotid artery intima-media thickness appears to be inversely correlated with serum lutein concentration [109]. Armoza et al. [47] studied the mechanisms involved in the action of tomato products in endothelial cells and demonstrated that inhibition of NF-*κ*B signaling may be one of the main mechanisms employed by lutein and lycopene to reduce inflammatory leukocyte adhesion to endothelium.

Herrero-Barbudo et al. [26] reported that the regular consumption of lutein is associated with an increase of the capacity of DNA repair in lymphocytes and a major resistance of DNA to endogenous damage. Nevertheless, a real correlation between the consumption of lutein and protection of DNA, proteins, and lipids from oxidative damage has not been demonstrated yet [107].

### 2.2. Vitamins

Tomato protective action is commonly attributed to the tomato antioxidant lycopene. However, tomato products are also sources of other compounds such as vitamins A, B, and E. Also these compounds have important effects on human health that will be described below.

#### 2.2.1. Vitamin E

Vitamin E family includes eight molecules: *α*-, *β*-, *γ*-, and *δ*-tocopherol; and *α*-, *β*-, *γ*-, and *δ*-tocotrienol. This classification is based on the nature of the isoprenoid chain; tocopherols contain a phytyl chain while tocotrienols contain a geranylgeranyl chain. These molecules are potent liposoluble antioxidant. Tocopherols are the most abundant vitamins in tomato plant and contribute to maintain optimal plant photosynthesis rate under high levels of stress [60]. Frusciante et al. [3] reported a vitamin E level in tomatoes between 0.17 and 0.62 mg/100 gr FW. Zanfini et al. [110] studied the antioxidant activity of lipophilic extracts obtained from different tomato varieties. They demonstrated a high synergistic effect of *α*-tocopherol-lycopene mixtures, and also for lycopene-*β*-carotene, lycopene-lutein, and lutein-*β*-carotene mixtures. This effect could be due to the fact that electrons can transfer from the carotenoid to the *α*-tocopherolxyl radical to regenerate *α*-tocopherol. An analogous mechanism has been suggested for the interactions between vitamin C, carotenoids, and *α*-tocopherol [111]. A synergistic interaction between ascorbic acid and *α*-tocopherol during the process of lipid peroxidation is well known [112]. This synergy was also observed during inhibition of the inflammation process [17].

Decreasing risk of advanced prostate cancer was associated with increasing dose of supplemental vitamin E uptake among men in the screening arm of the Prostate, Lung, Colorectal, and Ovarian Cancer Screening Trial [29].

In a study carried out on Finnish men and women, those who ingested higher intake of dietary vitamin E showed a decreased incidence of type 2 diabetes [30]. On the contrary, in a trial with a 10-year followup, with alternate-day doses of 600 IU (international units) vitamin E, there was not a significant benefit for type 2 diabetes in initially healthy women [113]. Similar results were reported for the cardiovascular events and mortality in healthy women that ingested 600 IU of natural-source vitamin E [114]. However, a study that evaluated the effect of adding tomato extract to the treatment regime of moderate hypertensives with uncontrolled blood pressure levels indicated a significant correlation between systolic blood pressure values and level of antioxidant activity [115].

#### 2.2.2. Vitamin C

L-ascorbic acid and dehydroascorbic acid are the main dietary forms of vitamin C, a labile molecule with reducing property. It is a water-soluble compound easily absorbed but it is not stored in the body. Abushita et al. [28] reported that salad tomatoes grown in field conditions contained between 15 and 21 mg/100 g FW, while a range of industrial grades of tomatoes had a mean value of 19 mg/100 g FW. Frusciante et al. [3] reported a vitamin C content between 8.0 and 16.3 mg/100 g FW.

Ascorbic acid (AsA) content in fresh tomatoes depends on genotype, climatic conditions, fruit development, maturation, senescence, and time of storage. AsA content in tomato fruit increases reaching a maximum and then began to decline with ripening [116]. Yahia et al. [117] reported a maximum level of 94.9 mg/100 g after 74 days from fruit set and slow reducing of its content with the color change. This decrease is concomitant with the increase in the activity of ascorbate oxidase, a Cu-containing enzyme that catalyses the oxidation reaction of ascorbate to DHA with the reduction of molecular oxygen to water. Adults have a mean body pool of 1.2–2.0 g of ascorbic acid that may be maintained with 75 mg/day of ascorbic acid [118]. Insufficient intakes of ascorbic acid lead to scurvy, a disease characterized by dry skin, open sores on the skin, weariness, impaired wound healing, and depression [119, 120].

Most of the known functions of ascorbic acid are correlated with its ability as electron donor and potent antioxidant in humans. In fact, vitamin C protects against oxidation of LDL by different types of oxidative stresses and inhibits LDL oxidation by vascular endothelial cells [120]. Yogeeta et al. [121] demonstrated that the combination of ferulic acid and ascorbic acid offered a protection to the myocardium. This was due to the attenuation of the alterations in the levels of lipids, lipid peroxidation, lipoprotein profile, and lipid metabolizing enzymes. Ascorbic acid with vitamin E prevents oxLDL-induced overexpression of vascular endothelial growth factor (VEGF) and its receptor is responsible for atherosclerotic plaque formation. In addition, vitamin C decreases plasma vascular cell adhesion molecule-1 responsible for monocyte adhesion and inflammation [31, 32].

Vitamin C in tomato is highly bioavailable, so a regular intake of small amounts of tomato products can increase cell protection from DNA damage induced by oxidant species. This effect may originate from the synergism of vitamin C with lycopene [122]. Some studies reported that ascorbic acid can prevent cancer by neutralizing free radicals before they can damage DNA and initiate tumor growth or may act as a prooxidant helping body to destroy tumors in their early stages [123]. Other researchers affirmed that there was no sufficient evidence that vitamin C and vitamin E can help prevent cancer [124].

Jacob et al. [33] discovered that the consumption of tomato juice (500 mL) for 2 weeks reduced total cholesterol and CRP (C-reactive protein) levels which is a marker of inflammation. The effect was stronger when vitamin C was added in a high dose. This study confirmed that a synergic effect between vitamin C and lycopene is required for the beneficial effects of tomato juice on oxidative stress and inflammation.

#### 2.2.3. Folates

Folates represent all forms of vitamin B found in biological systems, while folic acid is the synthetic form found in dietary supplements and fortified foods [125]. Iniesta et al. [126] investigated the level of 5-methyltetrahydrofolate in commercial raw tomato cultivar harvested in Murcia (Spain). They found the maximum level in Ronaldo cultivar, equal to 31.5 *μ*g/100 g FW.

Folate metabolism is involved in several physiological mechanisms in the field of andrology and gynecology [127]. In particular, folates have a role in various one-carbon transfer reactions, including purine and pyrimidine biosynthesis, amino acid metabolism, methylation of nucleic acids, proteins, and lipids [128]. For these reasons, folates are essential for fetal growth [129]. Folic acid supplementation (200–400 *μ*g/day) is recommended for pregnant women to reduce pregnancy-induced folate deficiency, which can lead to megaloblastic anemia [34].

Homocysteine metabolism is regulated by the nutritional status of folate, vitamin B-12, and vitamin B-6 [130]. Some researchers consider hyperhomocysteinemia a marker for cardiovascular disease or a risk factor [35, 131, 132].

### 2.3. Phenolics

Phenolic compounds are widespread phytochemicals composed of an aromatic ring and one or more hydroxyl substituents. They can be constituted by simple phenolic molecules or polymerised compounds [133, 134]. In tomato fruit, phenolics include flavonoids, phenolic acids (hydroxybenzoic and hydroxycinnamic acids), and tannins. Polyphenols are effective free radical scavengers mediated by* para*-hydroxyl group. Phenolics may modulate cellular signaling processes during inflammation or may serve as signaling agents themselves [135, 136]. The level and the composition of phenolic compounds in tomato fruits depend greatly on genotype, environmental, and storage conditions [137]. In particular. Luthria et al. [138] demonstrated that the spectral quality of solar UV radiation significantly affects phenolic content.

Polyphenolic compounds are associated with therapeutic tools in inflammatory diseases including cardiovascular diseases, obesity and type II diabetes, neurodegenerative diseases, cancer, and aging.

These effects are due to the phenolic ability to interact with a wide spectrum of molecular targets central to the cell-signaling machinery. Main molecular mechanisms include (a) the inhibition of proinflammatory enzymes, such as cyclooxygenase (COX-2), lipoxygenase (LOX), and inducible nitric oxide synthase (iNOS); (b) the inhibition of phosphoinositide 3-kinase (PI 3-kinase), tyrosine kinases, and nuclear factor-kappa B (NF-*κ*B); (c) the activation of peroxisome proliferators-activated receptor gamma (PPAR *γ*); and (d) the activation of mitogen-activated protein kinase (MAPK), protein kinase C (PKC), and the modulation of several cell survival/cell-cycle genes [48, 49].

Studies reported that resveratrol increases the survival of mice fed with a high caloric diet producing changes including improvement in insulin sensitivity and decreased fat accumulation and body weight. These changes are associated with induction of mitochondrial-related transcription factors such as estrogen-related receptor *α* (ERR-*α*), nuclear respiratory factor 1 (NRF-1), and transcription mitochondrial factor A (TFAM). Potential beneficial effects of polyphenols have also been obtained for quercetin, able to reduce FFA levels, total cholesterol, and body weight in rats [139].

Finally, in 2007 Shen et al. [140] conducted a human clinical trial to examine plasma antioxidation and the levels of blood lipids, after ingestion of tomato products. The authors demonstrated that triglyceride levels and low-density lipoprotein cholesterol were decreased, while high-density lipoprotein cholesterol was increased.

#### 2.3.1. Flavonoids

Flavonoids constitute the largest group of naturally occurring phenolics in tomatoes and contribute to the determination of aroma, fragrance, and colour [141, 142]. Main classes in tomatoes include flavonols (such as quercetin and kaempferol), flavanols (such as catechins), flavanones (such as naringerin), anthocyanidins, and stilbenes (such as resveratrol). They are usually located in the skin and only in small quantities in the other parts of the fruit. Tomatoes of the Spanish cherry variety Paloma reached levels of 203 *μ*g quercetin/g FW [143]. Detected levels of total flavonoids in tomatoes from commercial greenhouses in close proximity to Særheim Research Center were between 2.6 and 25.6 mg/100 FW [144].

Some flavonoids such as rutin (a flavonol glycoside), quercetin, resveratrol, and catechin are active in rheumatoid arthritis. The mechanisms of action include inhibition of osteoclast/macrophage differentiation and function and estrogen modulation [50, 53].

Rutin, quercetin, glycosides of quercetin, and resveratrol have been shown to exert intestinal anti-inflammatory activity [37, 38]. In particular, rutin and quercetin have been proposed to act as quercetin prodrugs, preventing premature absorption of the aglycone in the small intestine and releasing it in the colon [51, 52]. Quercetin exerts significant anti-inflammatory effects on IL-1*β* activated human astrocytes, reducing cytokines and chemokines and oxidative stress [61].

Resveratrol acts on blood lipid levels and also at the atherosclerotic plaque and reduces cellular infiltration, fibrosis, and expression of inflammatory cytokines in a model of autoimmune myocarditis [54, 55].

Rossi et al. [145] conducted an epidemiological study in Italy that demonstrated a favorable role of dietary proanthocyanidins on gastric cancer risk, while Nishiumi et al. [39] reported that flavonoids modulate signal transduction pathways at each stage of carcinogenesis. After absorption, flavonoids are transported to target organs where they exert their anticarcinogenic activity.

Anthocyanins are one of the flavonoid phytopigments; they may act on adipocytes and modulate the expression levels of adipocytokines. In particular, C3G (cyanidin 3-glucoside) was reported to upregulate the expression of adiponectin that can increase insulin sensitivity at the level of human adipocytes controlling obesity [56, 57]. In human endothelium, anthocyanins can inhibit TNF*α*-induced inflammation through monocyte chemoattractant protein-1 [40]. In animal models, intake of anthocyanin fruit extracts improves cognitive and motor performance [146, 147].

#### 2.3.2. Phenolic Acids

Phenolic acids are responsible for the astringent taste of vegetables [148]. They include hydroxybenzoic acids and hydroxycinnamic acids. Hydroxybenzoic acids are gallic, p-hydroxybenzoic, protocatechuic, syringic, and vanillic acids, while ferulic, caffeic,* p*-coumaric, and sinapic acids belong to hydroxycinnamic acids [134]. Chlorogenic acid is the most abundant in tomatoes. Martínez-Valverde et al. [149] analyzed tomatoes purchased in a local supermarket (Murcia, Spain). They reported amounts of chlorogenic acid between 14.31 (in Daniella variety) and 32.84 mg/kg FW (in Senor variety), caffeic acid between 1.39 (in Senor variety) and 13.00 (in Ramillete variety),* p*-coumaric acid between values under limit of detection (in Liso and Remate varieties) and 5.77 mg/kg (in Daniella variety), and ferulic acid between 1.60 (in Riso variety) and 5.38 (in Durina variety) mg/kg FW.

Luthria et al. [138] identified amounts of* p*-coumaric acid between 3.5 and 5.5 mg/100 g FW, while the content of ferulic acid was between 0.9 and 1.5 mg/100 g FW.

Olthof et al. [150, 151] reported that half of the ingested chlorogenic acid was metabolized to hippuric acid. Therefore the colonic microflora transforms dietary phenols into metabolites that reach the circulation.

However, phenolic acids are studied mainly for their role as antioxidants [62, 63]. Some studies reported a protective effect of caffeic acid and its related catechols against hydroxyl radical formation* in vitro *[64]. Lodovici et al. [41] investigated the effect of phenolic acids on oxidative DNA damage* in vitro*. They found that these compounds protected at low concentration* in vitro* against DNA oxidation induced by iron. Caffeic acid can block the biosynthesis of leukotrienes that are involved in immunoregulation. In addition, it was shown that caffeic acid possesses antitumor activity in humans [42]. Other studies reported a correlation between a series of phenolic acids with the inhibition of AP-1 transcriptional activity, implicated in the processes that control inflammation, cell differentiation, and proliferation [58].

Cinnamic acids exhibit strong antioxidant properties. 1,5-Dicaffeoylquinic is a hepatoprotector when challenged by carbon tetrachloride. This mechanism involves, among others, radical scavenging [65].

#### 2.3.3. Tannins

Tannins include compounds of hydrolysable tannins that are polymers of ellagic acid, or gallic and ellagic acids, with glucose and condensed tannins (proanthocyanidins), which derive from the condensation of monomers of flavanol units [152, 153]. They play an essential role in sensory properties of fruits and fruit products such as taste and color. Their antioxidant ability is exerted by scavenging free radicals, chelating trace metals, and binding proteins [66]. Some studies showed that tannins can enhance glucose uptake and inhibit adipogenesis, acting like potential drugs for the treatment of noninsulin dependent diabetes mellitus [43]. Koleckar et al. [44] reported that tannins, in addition to anti-inflammatory effects, have a role in the mechanisms of action of antibacterial, antiviral, anticarcinogenic, and cardiovascular system preventing.

## 3. Effects of Processing on Tomato Chemical Composition

Tomatoes are consumed fresh or are used to manufacture a wide range of processed products that can show a very different composition compared to fresh fruit. For example, significant losses of ascorbic acid can occur during postharvest storage period and during preparation and cooking of foods. This is due to oxidation and leaching into the water used for cooking [75, 154].

For the production of tomato paste from fresh tomatoes, both homogenization and heat treatment are used. Heat treatment and/or homogenization can disrupt the cellular matrix of tomatoes determining the bioavailability of different nutrients [155]. For example, retention of vitamin C improves with mild treatments and low temperatures [156]. Lycopene bioavailability was found increased after heat-processed tomatoes compared to fresh tomatoes [157, 158]. Other studies demonstrated that thermal processing of tomato pulp at 130°C improved the* in vitro* bioaccessibility of lycopene [159, 160]. However, as bound antioxidants are released by processing, labile antioxidant compounds are destroyed simultaneously [28].

Veronica et al. [158] observed loss of vitamin C in heat-processed tomatoes at 88°C with an estimated *D*_88_°_*C*_ value (the time taken for 90% reduction of the initial vitamin C content at 88°C) of 276 min. In addition, they found an antioxidant activity of raw tomatoes equal to 4.13 ± 0.36 *μ*mol of vitamin C equiv/g of tomato. After heat treatment at 88°C for 2, 15, and 30 min, the total antioxidant activity significantly increased to 5.29 ± 0.26, 5.53 ± 0.24, and 6.70 ± 0.25 *μ*mol of vitamin C equiv/g of tomato, respectively (*P* < 0.01). This effect could be explained by the increased amount of lycopene, the major phytochemical in tomatoes.

Gahler et al. [161] investigated how heat treatments affect the contents of vitamin C and polyphenols as well as the hydrophilic antioxidant capacity. Tomato juice was produced under industrial-like conditions; baked tomatoes as well as tomato sauce and tomato soup were prepared under household conditions. Vitamin C contents decreased during the thermal processing, while the total phenolics concentration and the water-soluble antioxidant capacity increased.

Seybold et al. [162] investigated the content of carotenoids and vitamin E in samples of tomato sauce, tomato soup, baked tomato slices, and tomato juice taken at different times of heating. *β*-Carotene amount decreased or was stable while *α*-tocopherol content significantly rose during short-term heating.

Chang et al. [163] studied the effects of hot-air-drying (AD) treatment on tomato. They found that this process could enhance the nutritional value of tomatoes by increasing parts of the total flavonoids, total phenolics, and lycopene contents.

Patras et al. [164] reviewed the effect of thermal processing on content of anthocyanins. A combination of unit operations involving heat such as blanching, pasteurization, and duration affected the anthocyanin content of fruits and vegetables. Cyanidin-3-glucoside and pelargonidin-3-glucoside in fruit puree were significantly affected by thermal process treatments of 70°C during holding times of 2 min.

As a whole, these evidences could have a significant impact on consumer's food selection, increasing their awareness of the health benefits of processed tomatoes in the prevention of chronic diseases and inflammation.

## 4. Biofortification

The ability of various tomato phytonutrients to be positively correlated to prevent or ameliorate chronic disease is appealing. The studies carried out on the effects of phytochemicals on human health prompted researchers to find novel ways to get biofortified tomato genotypes with enhanced levels of phytonutrients such as anthocyanins, lycopene, ascorbic acid, and folate. In the last few years, crop biofortification gave an enormous contribution to understand the relationship between diet and health, reduce the risk of chronic disease, and better understand the regulatory systems in plant species. As described below, attempts to create novel tomato plant lines that carry genes for the accumulation of essential nutrients in tomato fruit can be achieved both through metabolic engineering and conventional breeding [165, 166] (Table 3).

### 4.1. Metabolic Engineering

Plant metabolic engineering adopted different biochemical approaches to improve the amount of various phytonutrients in tomato fruits. One strategy is based on modification of key-rate limiting steps in metabolic pathways. For example, the expression in tomato of two isoforms of the GDP-mannose-3,5′-epimerase, a key enzyme of the ascorbic acid biosynthesis pathway, resulted in a modest increase of ascorbate (1.6-fold) in the fruits of transformed plant [167]. In another study, the cytosolic-targeted tomato* Mdhar* (monodehydroascorbate reductase) and* Dhar* (dehydroascorbate reductase) genes were overexpressed in tomato var. Micro-Tom. In the DHAR overexpressing lines, there was a 1.6-fold increase of AsA in fruits of plants grown in low light condition, while MDHAR transformants showed a reduced level of AsA by 0.7-fold in mature green fruits [168]. Another approach in metabolic engineering is the expression of genes of a specific biosynthetic pathway belonging to other organisms. For example, attempts to increase AsA accumulation in tomato fruits were carried out by expression of the genes* phytoene desaturase (crtI) *of* Erwinia uredovora *and the yeast-derived* GDP mannose pyrophosphorylase *(GMPase) and* arabinono-1,4-lactone oxidase *(ALO) [169, 170]. This strategy was also used to improve folate accumulation in fruit. The synthesis of tetrahydrofolate in mitochondria requires* hydroxymethyl dihydropteridine* (HMDHP), synthetized in the cytosol by GTP, and* p-aminobenzoate* (PABA), synthetized in chloroplasts from chorismate [171]. The first studies aimed to increase folate in tomato involved the overexpression of the bacterial GTP cyclohydrolase I (GCHI) to increase the supply of pteridines from cytosol. The transformed plants showed a low increase of folates (2- to 4-fold) in fruit probably because the supply of PABA became limiting [165, 172, 173]. The enhanced expression of a gene encoding for the aminodeoxychorismate synthase from* Arabidopsis* (AtADCS), aimed at increasing the supply of PABA, did not result in any change of folate levels in tomato fruit. On the contrary, the expression of GCHI and AtADCS resulted in 20-fold higher levels of folate compared to the control [171]. Using an alternative strategy, Apel and Bock [174] introduced the* lycopene β-cyclase *gene from the* Eubacterium Erwinia herbicola *and the higher plant* Narcissus pseudonarcissus* into the tomato plastid genome and obtained a 50% increase in total carotenoid accumulation in genotypes expressing the plant enzyme. Other studies involve the accumulation of novel metabolites in tomato, such as resveratrol, using the expression of a gene encoding for a grape stilbene synthase [175]. Another approach to improve the levels of phytonutrient in plant organs consists in modulating regulatory genes whose products control flux through several biosynthetic pathways. For example, Davuluri et al. [176] enhanced the amount of anthocyans by suppressing an endogenous photomorphogenesis regulatory gene,* DET1 (De-etiolated 1)*, using fruit-specific promoters combined with the RNA interference (RNAi) technology. The transformed plants showed an increase of flavonoid levels up to 3.5-fold. The last approach to increase levels of phytonutrients involves an enhancing expression of transcription factors that regulate specific biosynthetic pathways. This system does not work well in a complex branched pathway like those of folate biosynthesis [165] but seems to be very efficient to enhance the levels of secondary metabolites such as anthocyanins [177] and flavonols [184]. The anthocyanin pathway was modified in tomato using the transcription factors* Delia* (*Del*) and* Rosea1* (*Ros1*) from snapdragon* Antirrhinum majus*. The fruit of transformed plants accumulated high level of anthocyanins with concentrations similar to those found in blackberries and blueberries [177]. Another transcription factor (*AtMYB12*), which regulates the caffeoylquinic acid and flavonol synthesis, proved to be a good candidate for the production of tomato fruits with high levels of polyphenolic antioxidants compounds [184]. Drawbacks of this strategy are that not all the transcription factors regulating pathway have been identified and that transcriptional regulators could have pleiotropic effects when their expression is enhanced [165, 177, 185].

### 4.2. Plant Breeding

One way to produce biofortified plants is to take advantage of the natural variation of plant genomes. For example, recently a new genetic combination was obtained through conventional breeding, which produced a phenotype with deep purple fruit pigmentation, due to an accumulation of anthocyanins on the peels. Such a genotype was named Sun Black [178]. Tomato fruit quality usually exhibits quantitative variation controlled by several genes and influenced by environment. For these reasons one main focus of genetic studies has been the identification of genes and quantitative trait loci (QTL) that control the accumulation in tomato fruit of phytonutrients, such as lycopene [165, 178, 179]. As for other crops, the improvement of tomato depends on the availability of genetic variability. However, the germplasm of cultivated tomato shows a reduced genetic variability, both for its natural reproduction systems (autogamy) and as a consequence of domestication and breeding, the latter often based on intercrossing advanced lines. In contrast, wild species (such as* S. pimpinellifolium, S. neorickii, S. habrochaites, S. chmielewskii, *and* S. pennellii*) and heirlooms tomatoes have a rich genetic variability; therefore, one goal of plant breeding has become to screen wild genetic resources for valuable traits that could be introduced into modern varieties to improve specific traits [180, 186, 187]. In this regard, it is noteworthy that the work of Schauer et al. [187], in order to identify components of fruit metabolic composition, has phenotyped tomato introgression lines (IL) in which marker-defined genomic regions of the wild species* Solanum pennellii* were replaced with homologous intervals of the cultivated variety M82. In our laboratory, two introgression lines of* Solanum pennellii* were identified harboring QTLs that increase the content of ascorbic acid, phenols, and soluble solids [181, 182]. In a subsequent work, the selected QTLs were pyramided into cultivated varieties to increase antioxidant content in tomato fruits [183]. Although the conventional breeding is a good strategy to improve the quality of tomato fruit, this technique has some limitations due to sexual incompatibility and requires long breeding programs, whereas genetic engineering has no such limitations and novel genes can be introduced directly into local cultivars. Today, several genomic resources are available for tomato, such as high-density genetic maps, genomic and cDNA libraries, chip Illumina, germplasm collections, and populations of introgression lines [188]. In addition, the sequence of the tomato genome has recently been completed (http://solgenomics.net/organism/solanum_lycopersicum/genome) by an international consortium [189]. Using the existing genetic resources and genomic tools, it is now possible to integrate and apply the information of the genome sequence to discover new genes and allelic variants for genetic traits important for consumers, such as fruit quality. One of the most likely outcomes of the availability of the tomato genome sequence will be the development of high-throughput molecular markers to be used for genetic analysis and breeding programs and the discovery of novel genes. Moreover, by combining the sequence of tomato genome with the possibility of massive resequencing, it is now possible to investigate the genetic variability in large populations of individuals with the aim of identifying polymorphisms (SNPs/indels) in a panel of candidate genes. Finally, the use of approaches, such as RNA-seq, also called “Whole Transcriptome Shotgun Sequencing” (“WTSS”), provides a new gateway to identify the level of gene expression, new transcripts, splice variants, and expressed SNPs [190]. In Table 4, the main advantages and disadvantages of metabolic engineering and plant breeding are summarized.

## 5. Conclusion

There are many evidences supporting the anti-inflammatory and anticancer action of tomato fruit bioactive compounds. Molecular mechanisms controlling these effects have been extensively studied and described.

The present review reports a brief description, even though not exhaustive, of bioactive compounds available in tomato fruit and of their beneficial properties on human health.

The content of these compounds might be increased to obtain biofortified food taking into account, among other things, the great influence of processing transformation that is required for some derived tomato foods. Various strategies might be used to increase these compounds in tomato fruit. Therefore, the final destination of biofortified tomatoes needs to be considered when selecting new genotypes for fresh market or processing.

It is necessary to dissect tomatoes complex genetic control to identify key genes of biochemical pathways underlying their biosynthesis.

The best strategy to transfer them into improved genotypes might be chosen between metabolic engineering via genetic transformation and precision breeding by the aid of molecular markers.

In both cases, the richness of genetic and genomic resources today available for tomato might highly enhance the success of tomato breeding aimed to obtain new biofortified genotypes.

## Figures and Tables

**Table 1:** Effects of bioactive compounds occurring in tomato.

Class	Compound	Main effects	References
Carotenoids	Lycopene	Control of oxidative stress and inflammation (production of IL-10 and inhibition of IL-6 and IL-8)	[14–17]
		Cancer inhibition (prostate, breast, colorectal, endometrial, lung, oral, and pancreatic)	[18, 19]
	*β*-Carotene	Prevention of photooxidative damage	[20]
		Inhibition of atherosclerosis and prevention of myocardial infarction	[21]
	Lutein	Preservation of eye health and improvement of symptoms in ARMD	[22, 23]
		Protection against cardiovascular diseases (inhibition of NF-*κ*B signaling)	[24, 25]
		Increasing of DNA resistance to endogenous damage and repair	[26]

Vitamins	Vitamin E	Inhibition of lipid peroxidation and cardiovascular diseases	[27, 28]
		Decreasing risks of type 2 diabetes and advanced prostate cancer	[29, 30]
	Vitamin C	Inhibition of LDL oxidation and monocyte adhesion	[31, 32]
		Decreasing of total cholesterol and CRP	[33]
	Folates	Prevention of megaloblastic anemia in pregnant women and regulation of fetal growth	[34]
		Control of homocysteine metabolism	[35]
		Reduction in the rate of neural tube defects	[36]

Phenolic compounds	Flavonoids	Intestinal anti-inflammatory activity	[37]
		Inhibition of TNF*α*-induced inflammation	[38]
		Prevention of gastric cancer risk	[39, 40]
	Phenolic acids	Protection against DNA oxidation and antitumor activity against colon carcinogenesis	[41, 42]
	Tannins	Inhibition of adipogenesis	[43]
		Antibacterial, antiviral, anticarcinogenic, and cardiovascular action	[44]

**Table 2:** Main anti-inflammatory and antioxidant effects of bioactive tomato compounds.

	Compound	References
Anti-inflammatory effect	Lycopene	[14, 16, 17]
	*β*-Carotene	[45]
	Lutein	[46, 47]
	Vitamin E	[17]
	Vitamin C	[33]
	Rutin	[48–51]
	Quercetin	[48, 49, 52]
	Glycosides of quercetin	[53]
	Catechin	[50]
	Resveratrol	[37, 54, 55]
	C3G (cyanidin 3-glucoside)	[56, 57]
	Phenolic acids	[58]
	Tannins	[44]

Antioxidant effect	Lycopene	[15, 59]
	*β*-Carotene	[20, 59]
	Lutein	[46]
	Vitamin E	[27, 28, 60]
	Vitamin C	[31, 32]
	Polyphenols	[58]
	Quercetin	[61]
	C3G (cyanidin 3-glucoside)	[40]
	Phenolic acids	[41, 62–64]
	Cinnamic acids (1,5-dicaffeoylquinic)	[65]
	Tannins	[66]

**Table 3:** Results of biofortification through metabolic engineering and breeding in tomato.

Approach	Technique	Gene	Pathway	References
Metabolic engineering	Modification of a key-rate limiting step	*GDP-mannose-3,5*′*-epimerase* *monodehydroascorbate reductase *(*Mdhar*)- *dehydroascorbate reductase* (*Dhar*)	Ascorbate	[167, 168]
	Heterologous gene expression	*Erwinia uredovora*-*phytoene desaturase (crtI)*	*β*-Carotene	[169]
		Arabidopsis-*arabinono-1,4-lactone oxidase (ALO)* Yeast-derived-*GDP mannose pyrophosphorylase *(GMPase)	Ascorbic acid	[170]
		Bacterial *GTP-cyclohydrolase I *(*GCHI*)	Ascorbic acid	[170]
		Arabidopsis-*Aminodeoxychorismate synthase* (*ADCS)*	Folate	[165, 171–173]
		*Erwinia herbicola lycopene b-cyclase*	Carotenoid	[174]
		Grape *stylbene synthase *	Resveratrol	[175]
	Silencing pathway bottle neck	*De-etiolated1 (DET-)1 *	Anthocyans	[176]
	Increase levels of transcription factors	*Delia* (*Del*) and *Rosea1* (*Ros1*) *AtMYB12 *	Anthocyans Polyphenols	[177] [177]

Conventional breeding	Association mapping QTL	29 SSR and 15 morpho-physiological traits	Anthocyans	[178]
	QTL linkage mapping	*Lyc 7.1* and *lyc 12.1 *	Lycopene	[179]
		Variation during the plant development	Various components	[180]
			Ascorbic acid, phenols, and soluble solid	[181, 182]
			Total phenols and °Bx content	[183]

**Table 4:** Advantages and disadvantages of metabolic engineering and plant breeding.

Metabolic engineering	Plant breeding
Advantages	
(i) Fast (ii) Easy to apply to the elite cultivar (iii) Genes from other organism can be inserted (iv) Genes are inserted directly into the plant genome and are transmitted to offspring (v) Possible selection of the expression organs	(i) It uses the natural genome variation of crop (ii) Few legal regulations to follow

Disadvantages	
(i) Many legal regulations regarding the landscape (ii) Political and socioeconomic disputes about the “transgenics”	(i) Restricted to the crop gene pool (ii) Restricted to the only sexually compatible plants (iii) It needs work for a long time and with a high number of generations to have plants with the genes of interest
